# Mitochondrial Toxic Effects of Antiepileptic Drug Valproic Acid on Mouse Kidney Stem Cells

**DOI:** 10.3390/toxics11050471

**Published:** 2023-05-20

**Authors:** Minsu Lee, Changhwan Ahn, KangMin Kim, Eui-Bae Jeung

**Affiliations:** 1 Laboratory of Veterinary Biochemistry and Molecular Biology, College of Veterinary Medicine, Chungbuk National University, Cheongju 28644, Republic of Korea; 2 Laboratory of Veterinary Physiology, College of Veterinary Medicine, Jeju National University, Jeju 63243, Republic of Korea; 3 Veterinary Medical Research Institute, Jeju National University, Jeju 63243, Republic of Korea

**Keywords:** mitochondria, toxic effects, valproic acid, kidney, stem cell

## Abstract

Valproic acid (VPA) is a histone deacetylase inhibitor that is used mainly as an antiepileptic and anticonvulsant drug. The side effects of VPA usually appears as hepatic injury and various metabolic disorders. On the other hand, it is rarely reported to cause kidney injury. Despite the many studies on the influence of VPA exposure on the kidneys, the specific mechanism remains unclear. This study examined the changes after VPA treatment to the mouse kidney stem cells (mKSCs). VPA triggers an increase in mitochondrial ROS, but there was no change in either mitochondrial membrane potential or the mitochondrial DNA copy number in mKSCs. The VPA treatment increased the mitochondrial complex III but decreased complex V significantly compared to the DMSO treatment as a control. The inflammatory marker (IL-6) and the expression of the apoptosis markers (Caspase 3) and were increased by VPA. In particular, the expression of the podocyte injury markers (CD2AP) was increased significantly. In conclusion, VPA exposure has adverse effects on mouse kidney stem cells.

## 1. Introduction

Valproic acid (VPA) is the most common drug for treating convulsions and epilepsy because of its powerful therapeutic effect against seizures, epilepsies, and migraine control [1]. VPA blocks the transcriptional action by inhibiting the activity of histone deacetylase class I. The drug can also alleviate spasms by inhibiting enzymes, such as GABA transaminase, succinate semialdehydrogenase, and alpha-ketoglutarate dehydrogenase [2]. When VPA enters the mitochondria, it binds to coenzyme A (CoA) to form VPA-CoA, which interferes with the metabolic pathway involving CoA, such as fatty acid oxidation [3]. The side effects of VPA include nausea, vomiting, diarrhea, progress, urticaria, arousing liver dysfunction, and kidney function abnormalities [4]. Exposure to VPA can cause urea cycle disorder that inhibits the release of ammonia into urea by inactivating the carbamoyl phosphate synthetase I involved in the production of carbamoyl phosphate. This process leads to hyperammonemia, which increases the concentration of ammonia in the blood, causes kidney toxicity, or accelerates kidney dysfunction [5].

Whether VPA causes nephrotoxicity is unclear, but the uric acid and creatine levels of rats that had received 100 mg/kg or 500 mg/kg of VPA daily for 30 days were higher than the controls, with a concomitant decrease in the activity of the antioxidant enzyme [6]. By contrast, Costalonga EC et al. (2016) reported that VPA had beneficial effects against ischemic reperfusion injury (IRI) in the kidney [7]. The acute tubular necrosis (ATN) grade was higher in IRI-induced rats than in normal rats, and the mRNA expression level of the inflammation markers (IL-6, TNF-α, and MCP-1) were increased in IRI-induced rat than in normal rat. On the other hand, when IRI-induced rats were treated with VPA, the ATN grade and mRNA expression levels of the inflammation marker returned to normal state.

Many alternative experiments have evaluated kidney toxicity in vitro [8]. Despite the in vitro experimental results using human embryonic stem cells (ESCs) and induced pluripotent stem cells (iPSCs) [9], there are disadvantages when introducing stem cells because they require an extensive protocol and have low sensitivity. Therefore, it is necessary to develop an in vitro model suitable for nephrotoxicity studies.

This study examined whether mouse kidney stem cells (mKSCs) are suitable for validating nephrotoxic substances. In addition, the pathophysiological alterations caused by VPA in the mKSCs, and the underlying mechanism were assessed.

## 2. Materials and Methods

### 2.1. Cell Culture

Mouse kidney stem cells were purchased from AcceGen (ABC-SC0037, Fairfield, CT, USA) and cultured in 3 mL of a MOUSE KIDNEY STEM CELL Growth Medium Kit (ABM-SM0037, AcceGen, NJ, USA) in a T-25 culture flask (70025, SPL, Pocheon, Republic of Korea). The cells were incubated in a 5% CO2 and 37 °C humidified tissue culture incubator (Sanyo, Osaka, Japan). The human embryonic kidney cells (HEK293F) were purchased from Invitrogen (Waltham, MA, USA) and were grown in DMEM (LM001-05, WELGENE, Gyeongsan, Republic of Korea) containing 10% FBS, 50 μg/mL plasmocin (InvivoGen, San Diego, CA, USA), 100 unit/mL penicillin, and 100 mg/mL streptomycin (L0022, Biowest, Nuaillé, Frances).

### 2.2. Measurement of Cell Viability

The mKSCs were seeded on 96-well plates with 7000 cells/well in 70 μL of MOUSE KIDNEY STEM CELL Growth Media. After incubation for 24 h, the drug media were added to 100 μL/well. After culturing in an incubator for 24 h, a CCK-8 assay was carried out using an EZ-cytox solution (EZ-500, DoGenBio, Seoul, Republic of Korea). All the remaining drug media were removed, and the cells were washed once with DPBS (LB001-02, WELGENE, Seoul, Republic of Korea). Subsequently, 110 μL of a mixture of pure EZ-cytox solution and DMEM at a ratio of 1:10 was added. The resulting mixture was incubated for one hour. After incubation, the absorbance at 450 nm was evaluated using a Synergy H1 microplate reader (Biotek instrument, Winooski, VT, USA). The cell viability was evaluated by comparing the relative absorbances with the absorbance of the solvent control set to 100%. The IC50, which is indicative of 50% cell viability, was substituted for the value of the *y*-axis in the trend line formula of the graph.

### 2.3. Measurement of mtROS

The drug treatment conditions were identical to those for measuring cytotoxicity. The level of mitochondrial ROS (mtROS) was measured 24 h after the VPA treatment. The cells were treated with mitoSOX dye (M36008, Invitrogen, MA, USA; 3 μM) and Hoechst 33342 (H3570, Invitrogen; 12 μg/mL) in pure DMEM for 10 min at 37 °C. The cells were washed twice with DPBS to determine the clear fluorescence absorption intensity using a Synergy H1 microplate reader (Agilent Technologies, Santa Clara, CA, USA).

### 2.4. Measurement of Mitochondrial Membrane Potential

The mitochondrial membrane potential (ΔΨm) was determined four hours after VPA treatment. The drug treatment conditions were identical to those when measuring the cytotoxicity. The cells were washed with DPBS and evaluated mitochondria membrane potential with JC^−1^ dye (T3168, Invitrogen; 5 μg/mL) for 10 min at 37 °C. The cells were then washed twice with DPBS to determine the fluorescence absorption intensity using a Lionheart FX (Agilent Technologies).

### 2.5. RNA Purification, cDNA Synthesis, and RT-PCR

Trizol (AM9738, Invitrogen) reagent was used for total RNA purification. Reverse transcription was performed for cDNA synthesis using 1 μg RNA, and the final volume was made up to 20 μL using an iScript™ cDNA Synthesis Kit (BR170-8891, Bio-rad, Hercules, CA, USA). The transcription levels of the genes were measured by RT-PCR (Quantstudio 3, applied biosystems, Waltham, MA, USA) using a Prime Q-Mastermix and ROX Dye (Q-9210, GENEBIO, Busan, Republic of Korea). RT-PCR was conducted under the following steps: pre-denaturation at 95 °C for 10 min, 40 cycles of denaturation at 95 °C for 30 s, annealing at 58 °C for 30 s, and extension at 72 °C for 30 s. The fluorescence intensity was measured at the extension phase of every single cycle. QuantStudioTM Design and analysis Software ver.1.4.1 was used for template design and ΔCt value analysis. The Expression levels of genes were compared quantitatively using the ∆∆Ct method with GAPDH as the reference gene. The average Ct values were obtained from the three wells. The sequences of primers and probes were as follows:

TGF-β (forward: 5′-CGTGGAAATCAACGCTCCAC-3′, reverse: 5′-CCCGGGTTGTGTTGGTTGTA-3′), IL-6 (forward: 5′-CCCCAATTTCCAATGCTCTCC-3′, reverse: 5′-CGCACTAGGTTTGCCGAGTA-3′), MMP-9 (forward: 5′-GCCCTGGAACTCACACGACA-3′, reverse: 5′-TTGGAAACTCACACGCCAGAAG-3′), Bcl-2 (forward: 5′-TAAGCTGTCACAGAGGGGCT-3′, reverse: 5′-TGAAGAGTTCCTCCACCACC-3′), Bax (forward: 5′-CGAGCTGATCAGAACCATCA-3′, reverse: 5′-GAAAAATGCCTTTCCCCTTC-3′), Casp3 (forward: 5′-GATAATGTCTTAGAACTTGAATCC-3′, reverse: 5′-CTTCCATAAATCAGGTCCAA-3′), CD2AP (forward: 5′-GCAGAAGCAGATGATGGGAA-3′, reverse: 5′-ACAGCTTTCTTCAGCTTTGC-3′), VEGF-A (forward: 5′-CAGGCTGCTGTAACGATGAA-3′, reverse: 5′-CCTTTCCCTTTCCTCGAACT-3′), Nephrin (forward: 5′-TCATATCGCCAAGCCTTCAC-3′, reverse: 5′-TCCCCTTGGGTCCTCATATT-3′), Podocin (forward: 5′-CCCAAGATGTAAAGGTTGCC-3′, reverse: 5′-TTTCCCCTTCTGCAGCAATC-3′), and POXDL (forward: 5′-AGGCTGGAGTCATCGACATT-3′, reverse: 5′-CTCTGTGAGTCGTTGTTGGT-3′).

### 2.6. Analysis of Mitochondrial Gene Expression and mtDNA Copy Number

Mitochondrial gene expression was measured by extracting the total RNA and synthesizing cDNA, followed by real-time polymerase chain reaction (RT-PCR), as mentioned above. For RT-PCR performance, 2 μL of cDNA was analyzed by RT-PCR. For mtDNA expression analysis, the total DNA was extracted using G-DEXTM-IIc (17231, iNtRON, Daejeon, Republic of Korea). The total DNA was diluted to 3 ng/μL, then 2 μL of total DNA was analyzed by RT-PCR, as described above. Mitochondrial Copy number is quantified by taking the ratio between a target mitochondrial gene and a reference nuclear gene (mtDNA/nDNA) using quantitative real-time PCR. β2-microglobin was used for the reference gene of nDNA. The sequences of primers and probes were as follows:

Complex I (forward: 5′-TTCTTGCAGCTGTGTCCAAC-3′, reverse: 5′-AGCATTTTGGGAGGGTTCTT-3′), Complex II (forward: 5′-ACACAGACCTGGTGGAGACC-3′, reverse: 5′-GGATGGGCTTGGAGTAATCA-3′), Complex III (forward: 5′-TGGTCTCCCAGTTTGTTTCC-3′, reverse: 5′-GCAGCTTCCTGGTCAATCTC-3′), Complex IV (forward: 5′-TGCTCAACGTGTTCCTCAAG-3′, reverse: 5′-TAAGGGTCCAAAACCAGTGC-3′), Complex V (forward: 5′-CGGACAGATGTCCTTCACCT-3′, reverse: 5′-ACTTAGTCGTGGTGCCGTCT-3′), SOD2 (forward: 5′-GAGTTGCTGGAGGCTATCAA-3′, reverse: 5′-CGACCTTGCTCCTTATTGAA-3′), Catalase (forward: 5′-TGATCTGACCAAGGTTTGGC-3′, reverse: 5′-CTGAAGCATTTTGTCAGGGC-3′), Mitochondrial DNA (forward: 5′-CCCAGCTACTACCATCATTCAAGT-3′, reverse: 5′-GATGGTTTGGGAGATTGGTTGATGT-3′), β2-microglobin (forward: 5′-TGTCAGATATGTCCTTCAGCAAGG-3′, reverse: 5′-TGCTTAACTCTGCAGGCGTATG-3′), and GAPDH (forward: 5′-TGGAAAGCTGTGGCGTGAT-3′, reverse: 5′-TGCTTCACCACCTTCTTGAT-3′).

### 2.7. Statistical Analysis

Significant differences were detected using one-way ANOVA followed by a Tukey’s test for multiple comparisons. The analysis was performed using the Prism Graph Pad v8.0.1 (Graph Pad Software, San Diego, CA, USA). The obtained values are expressed as the means ± SD of at least three separate experiments; *p* values < 0.05 were considered significant.

## 3. Results

### 3.1. Effects of Drugs on the mKSCs and HEK293F Cell Viability

**Figure 1:**
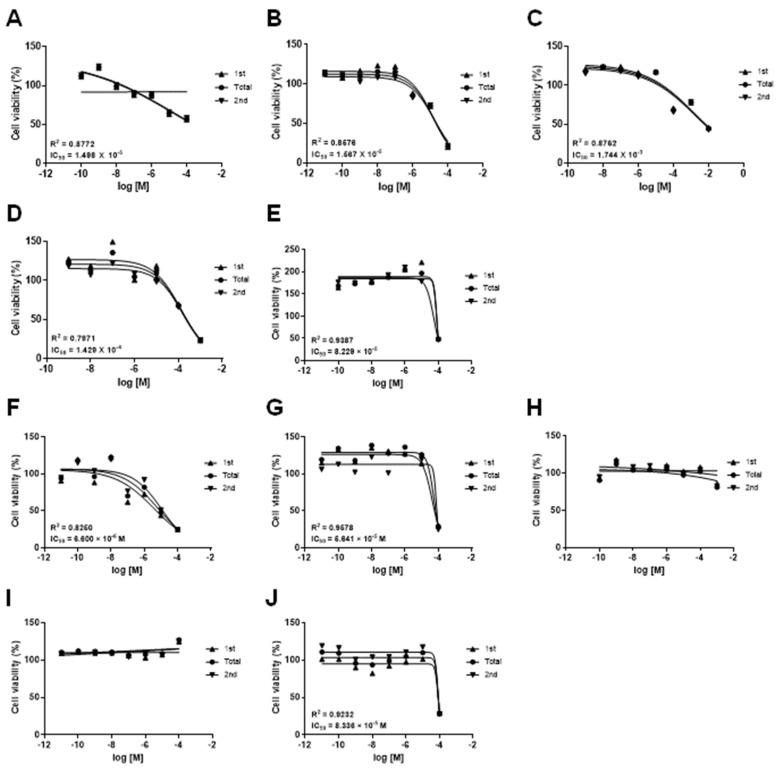
Effects of five drugs in mKSCs and in HEK293F. The mKSCs and HEK293F cytotoxicity assay were carried out. The cell viability was measured using a CCK-8 assay. Dose–response curve of the cell viability in mKSCs with (**A**) doxorubicin, (**B**) (Z)-4-hydroxytamoxifen, (**C**) acetaminophen, (**D**) valproic acid sodium salt, and (**E**) amiodarone. Dose–response curve of the cell viability in HEK293F with (**F**) doxorubicin, (**G**) (Z)-4-hydroxytamoxifen, (**H**) acetaminophen, (**I**) valproic acid sodium salt, and (**J**) amiodarone. The cell viability assays were performed twice for the validation of the data, it represents in the figures as first (1st), second (2nd), and mean of the data (total). Each value is expressed as the means ± SD. n = 6.

The mKSCs and HEK293F cells were exposed to the drug to find a cell line suitable for kidney toxicity. The drugs tested were doxorubicin, (Z)-4-hydroxytamoxifen, acetaminophen, valproic acid, sodium salt, and amiodarone. The five drugs caused mitochondrial DNA damage, inhibited mitochondrial-derived respiration, and disrupted the mitochondrial permeability transition pores (mPTP) [10,11,12,13,14]. As shown in the dose–response graph of Figure 1 and Table 1, doxorubicin, (Z)-4-hydroxytamoxifen, and amiodarone similarly decreased the cell viability in both mKSCs (Figure 1A,B,E) and HEK293F cells (Figure 1F,G,J). On the other hand, VPA and acetaminophen decreased the cell viability of only mKSCs (Figure 1C,D), not in HEK293F (Figure 1H,I). mKSCs react more to VPA and acetaminophen exposure than HEK293F cells. Acetaminophen can induce hepatotoxicity and nephrotoxicity by being metabolized into the toxic metabolite, N-acetyl-p-benzoquinone imine, in the liver. On the other hand, it is unclear if VPA induces nephrotoxicity. Therefore, the following experiment examined the basal mechanism for the kidney toxicity of VPA in mKSCs.

### 3.2. Effect of Drug on Mitochondrial Function in mKSCs and HEK293F

**Figure 2:**
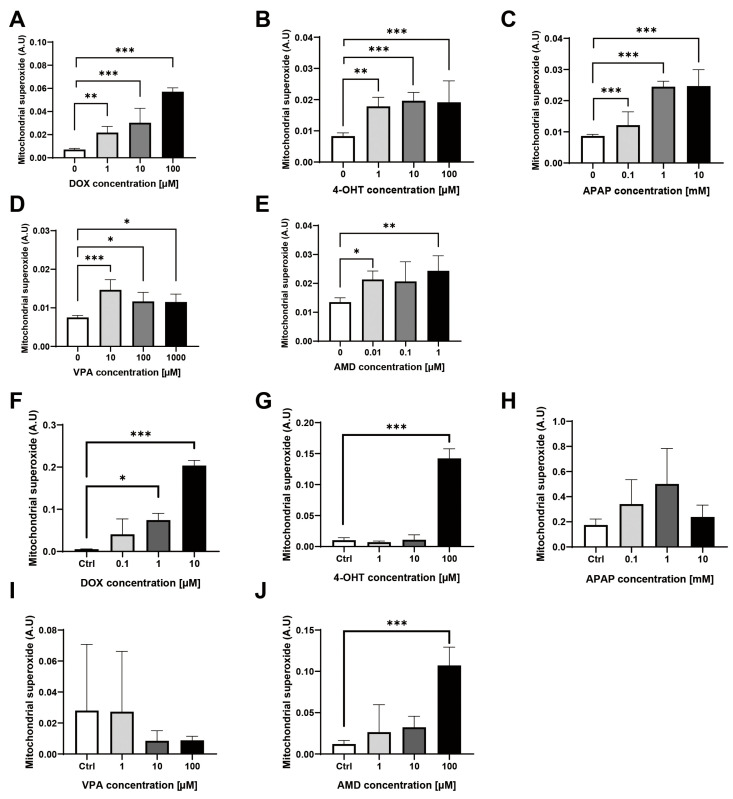
Changes in mitochondrial reactive oxygen species (mtROS) by five drugs in mKSCs and in HEK293F. Bar graph of mitochondrial superoxide using mitoSOX staining to measure a mtROS with (**A**) doxorubicin (DOX), (**B**) (Z)-4-hydroxytamoxifen(4-OHT), (**C**) acetaminophen (APAP), (**D**) valproic acid sodium salt (VPA), and (**E**) amiodarone (AMD) in the mKSCs. Bar graph of mitochondrial superoxide using mitoSOX staining to measure a mtROS with (**F**) doxorubicin, (**G**) (Z)-4-hydroxytamoxifen, (**H**) acetaminophen, (**I**) valproic acid sodium salt, and (**J**) amiodarone in the HEK293F cell line. The significance was obtained by ANOVA. * *p* < 0.05, ** *p* < 0.01, *** *p* < 0.01 vs. Control. Each value is expressed as the means ± SD. n = 6.

**Figure 3:**
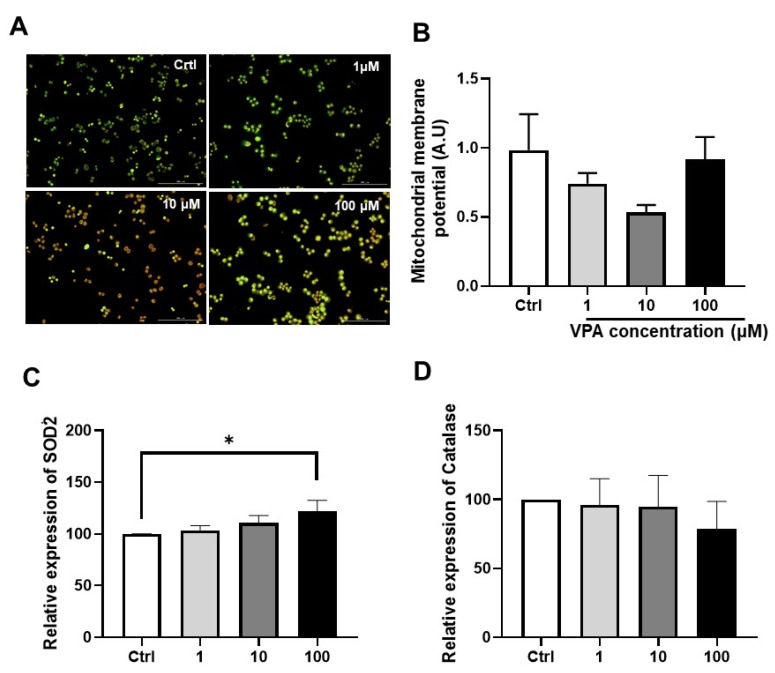
Change in the transcription level of SOD2 and catalase by the VPA treatment. (**A**) Representative picture of JC-1 staining to detect a mitochondrial membrane potential (MMP) with VPA treatment for 24 h in mKSCs treatment for 24 h in ABC cell and (**B**) Bar graph of MMP. The mKSCs cells were treated with VPA for 4 h, and gene transcription level was measured by real-time PCR. (**C**) Bar graph of Superoxide dismutase 2 (SOD2) and (**D**) Catalase. The significance was obtained by ANOVA. * *p* < 0.05 vs. Control. Each value is expressed as means ± SD. n = 3. Ctrl: Control; VPA; Valproic acid. The scale bar of the picture is 200 μm.

VPA is a typical mitochondrial toxic substance. Mitochondrial reactive oxygen species (mtROS) are markers to detect mitochondria dysfunction because the mitochondria are often injured under oxidative stress. In particular, the scavenging of mitochondrial-targeted ROS prevented podocyte loss, albuminuria, glomerulosclerosis, and kidney failure [15]. As shown in the mitochondrial superoxide graph of Figure 2, doxorubicin, (Z)-4-hydroxytamoxifen, and amiodarone increased the mtROS in both mKSCs (Figure 2A,B,E) and HEK293F cells (Figure 2F,G,J). On the other hand, VPA and acetaminophen increased the mtROS of only mKSCs (Figure 2C,D), not in HEK293F (Figure 2H,I). These results show the same tendency as the results of cell viability. No changes in the mitochondrial membrane potential were noted (Figure 3A,B), but the transcription levels of the SOD2 and catalase genes were measured by RT-PCR. SOD2 was upregulated by a 100 μM VPA treatment for four hours (Figure 3C), but catalase was unaffected (Figure 3D).

### 3.3. Effect of VPA on Mitochondrial Contents in mKSCs

**Figure 4:**
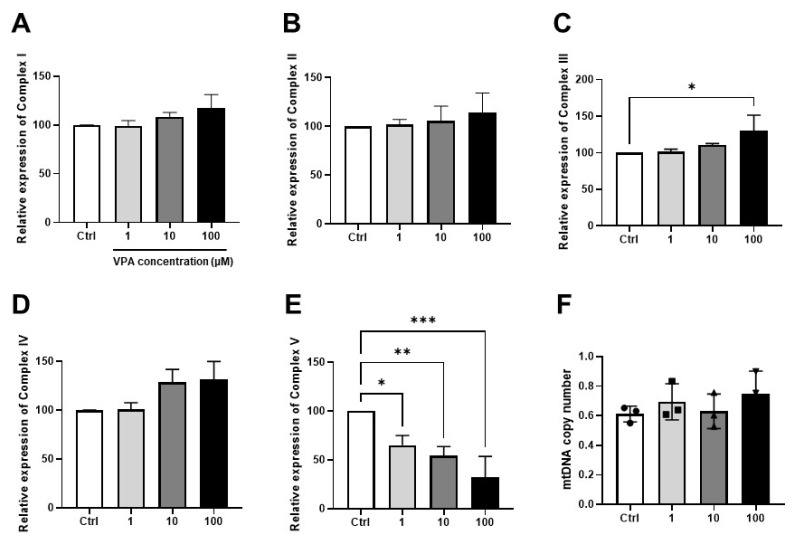
Effect of VPA on the mitochondrial contents. The mKSCs were treated with VPA for 4 h, and the gene transcription level was measured by real-time polymerase chain reaction (RT-PCR). (**A**) Bar graph of Complex I gene (NADH dehydrogenase); (**B**) Bar graph of Complex II gene (Succinate dehydrogenase); (**C**) Bar graph of Complex III gene (Coenzyme Q—cytochrome c reductase); (**D**) Bar graph of Complex IV gene (Cytochrome c oxidase); (**E**) Bar graph of Complex V gene (ATP synthase). (**F**) Bar graph of mitochondrial DNA copy number measured by RT-PCR in mKSCs cells with VPA treatment for 24 h. dot (●), square (■), triangle (▲), reversed triangle (▼) represent each data for mtDNA copy number for each sample. The significance was obtained by ANOVA. * *p* < 0.05 ** *p* < 0.01 and *** *p* < 0.001 vs. Control. Each value is expressed as means ± SD. n = 3. Ctrl; Control, VPA; Valproic acid.

The mitochondria are the key components of the generation and regulation of cellular bioenergetics. In addition, they produce most adenosine triphosphate (ATP) molecules using oxidative phosphorylation (OXPHOS) in the kidney. OXPHOS consists of five protein complexes: Complex I (NADH dehydrogenase), Complex II (Succinate dehydrogenase), Complex III (Coenzyme Q—cytochrome c reductase), Complex IV (Cytochrome c oxidase), and Complex V (ATP synthase). The five complexes were measured by RT-PCR after a four-hour VPA treatment. The mRNA level of Complex III was increased in the VPA 100 μM treatment group, and Complex V decreased depending on the VPA concentration (Figure 4C,E). On the other hand, there was no significant variation in the mRNA expression levels of the other mitochondrial complex genes, Complex I, Complex II, and Complex IV (Figure 4A,B,D). The VPA treatment did not change the mtDNA copy number used to measure the mitochondria density (Figure 4F).

### 3.4. Effect of VPA on mKSCs Inflammation, Apoptosis, and Podocyte Injury Marker

**Figure 5:**
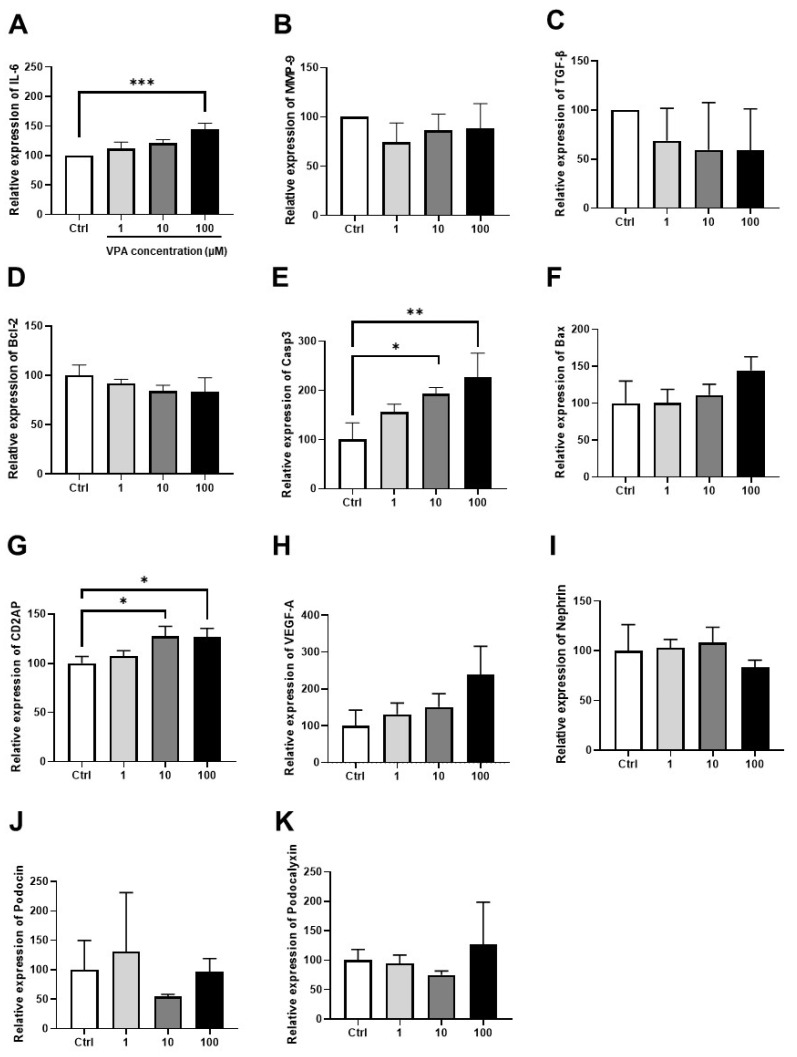
Effect of VPA on mKSCs inflammation, apoptosis, and podocyte injury marker gene expression levels. The cells were treated with VPA for 4 h in mKSCs, and mRNA expression was measured by real-time polymerase chain reaction (RT-PCR). The bar graphs of inflammation marker gene: (**A**) IL-6, (**B**) MMP-9, (**C**) TGF-β, of apoptosis-related gene: (**D**) Casp3, (**E**) Bcl-2, (**F**) Bax, and the podocyte injury marker gene: (**G**) CD2AP, (**H**) VEGF-A, (**I**) Nephrin, (**J**) Podocin, and (**K**) Podocalxyin. GAPDH gene expression was used as the reference gene. Significance was obtained by ANOVA. * *p* < 0.05, ** *p* < 0.01, and *** *p* < 0.001 vs. Control. Each value is expressed as means ± SD. n = 3. Ctrl: control, IL-6: Interleukin-6, MMP-9: Matrix metalloproteinase-9, TGF-β: Transforming Growth Factor-β; Bcl-2: B-cell lymphoma 2; Casp3: Caspase 3; Bax: Bcl-2 associated X; CD2AP: CD2 associated protein; VEGF-A: Vascular endothelial growth factor A.

Oxidative stress causes inflammation in the kidneys and decreases kidney function via apoptosis. After four hours of treatment with VPA, the mRNA expression level of IL-6, a kidney inflammatory marker, was increased significantly by the 100 μM VPA treatment (Figure 5A). On the other hand, there was no significant variation in the mRNA expression levels of the other inflammatory markers, MMP-9 and TGF- β (Figure 5B,C). In addition, the VPA treatment increase caspase 3 mRNA expression significantly, which is involved in apoptosis (Figure 5E), but there was no significant variation in the Bcl-2 and Bax mRNA expression (Figure 5D,F). These results show that VPA is involved in cell inflammation and apoptosis.

The podocyte injury marker was examined because mKSCs cells express podocyte-specific markers, such as the podocin, nephrin, and podocalyxin genes. After four hours of treatment with VPA, CD2AP mRNA expression was increased significantly in the 10 μM and 100 μM VPA treatment groups (Figure 5G). On the other hand, there was no significant variation in the mRNA expression levels of the other inflammatory markers, VEGF-A, nephrin, podocin, and podocalyxin (Figure 5H–K).

## 4. Discussion

Acute kidney injury (AKI) is defined by a rapid increase in serum creatinine, a decrease in urine output, or both [16]. AKI caused by drugs is a complex combination of the specific drug nephrotoxicity, metabolism, excretion, and patient characteristics [17]. Drugs, such as aminoglycoside antibiotics, amphotericin B, and cisplatin, cause renal tubular toxicity and paralyze mitochondrial function by causing oxidative stress [17,18,19]. AKI causes various metabolic changes that occur inside cells and in mitochondria. For decades, several mouse AKI models have been developed to examine the mechanism of AKI development, renal pedicle clamping (ischemia reperfusion injury), Cisplatin-induced nephrotoxicity, sepsis (LPS, cecal slurry, and cecal ligation and puncture), folic acid, and rhabdomyolysis are representative AKI model for study the disease mechanisms [20]. Funk JA et al. (2012) reported that the expression of mitochondrial respiratory proteins ATP synthase β and cytochrome c oxidase subunits I and IV were attenuated significantly in the myoglobinuria AKI model [21]. Furthermore, in the folic acid-induced mouse AKI model, mitochondrial DNA fragmentation occurred during excessive mitochondrial fission but before tubular cell death [22]. Hence, the mitochondria and the pathogenesis of AKI are closely related.

This study examined the effects of VPA on mouse kidney stem cells. The decrease in cell viability according to the VPA concentration was observed only in mKSCs cells, whereas HEK293F, commonly used in kidney cells, did not show any changes in cell viability. The cell viability results confirmed that mKSCs are a more sensitive cell to VPA than HEK293F. Therefore, mKSCs may be used more frequently than HEK293 cells if the substance is mildly toxic or cell-type specific. Due to stem cells exhibiting low mitochondrial content, mKSCs may be sensitive to mitochondria-toxic materials [23].

Sitarz et al. (2014) reported that VPA induces mitochondrial dysfunction in fibroblasts [24]. In this study, the mtROS level of mKSCs was increased by the VPA treatment. When mitochondrial oxidative stress is not adjusted, it eventually leads to cell death by disrupting the balance of the membrane potential generated in mitochondria or opening the mPTP [25]. The cells have mechanisms to minimize the generation of mtROS [26]. Superoxide dismutase 2 is a representative mitochondrial antioxidant enzyme. Interestingly, the mRNA level of SOD2 was increased in the 100 μM VPA treatment group. In a previous study, mRNA overexpression of superoxide dismutase caused an increase in the ROS level and oxidative stress [27]. This can be further evidence of oxidative stress induced by VPA exposure.

The changes in the mRNA level of the mitochondrial complexes by the VPA treatment were also confirmed. In particular, the expression level of mitochondrial complex V, an ATP synthase, decreased significantly with increasing VPA concentration. ATP synthase is involved in the final step of the oxidative phosphorylation of ATP production. A decrease in the ATP synthase mRNA expression level means that the amount of ATP produced by the cells may decrease. A previous study reported that the VPA-induced inhibition of mitochondrial biogenesis results in increased glycolysis [28], showing that similar action can occur in mouse kidney cells. In addition, the mRNA expression level of mitochondrial complex III was increased with increasing VPA concentration, which is associated with an increase in mtROS. According to previous studies, complex III is a major contributor to intracellular ROS generation and releases it into the inner membrane space and matrix of the mitochondria. Hence, an increase in mitochondrial complex III by VPA a treatment may cause mtROS, which participates in oxidative stress in mKSCs [29].

Mitochondrial dysfunction caused by oxidative stress induces host cell inflammation and, eventually, apoptosis [30]. As the VPA concentration was increased, the mRNA expression of IL-6, an inflammatory marker, increased, but there was no significant change in the mRNA expression of TGF-β and MMP-9. The mRNA expression level of Casp3, a representative apoptosis marker, increased significantly. Casp3 is an enzyme that is activated in the last stage of apoptosis [31], and the increase in Casp3 mRNA expression indicates that VPA can induce apoptosis in mouse kidney cells. Therefore, oxidative stress caused by VPA induces an inflammatory response in kidney stem cells that can also interfere with normal cell growth and inhibit cell function.

The level of CD2AP mRNA expression, a marker of podocyte damage, increased when the cells were treated with VPA for four hours. CD2AP is a protein that interacts with the enzymes involved in cytoskeletal remodeling, membrane transport, and cell motility in the slit diaphragm of podocytes. In vivo, podocytes form a slit diaphragm with other podocytes adjacent to each other and interact, such as material exchange. The slit diaphragm is broken when adjacent podocytes are damaged, and the area of the podocytes increases to form a slit diaphragm with other podocytes except for the damaged podocytes [32]. On the other hand, there were no significant changes in the mRNA expression level of the podocyte damage markers, such as nephrin, podocin, and VEGF-A, except for CD2AP. Nevertheless, the changes in the podocyte morphology and podocyte damage markers should be confirmed in vivo through histological analysis to prove that VPA damages podocytes.

This study examined the effects of VPA exposure on mouse kidney stem cells. VPA induced oxidative stress in kidney cells, which damages the mitochondria. In conclusion, VPA should be considered a nephrotoxic substance. In future research, examinations of VPA’s metabolic and epigenetic effects are needed. In vitro stem cell experiments have limitations for investigating the metabolic effect of target molecules, and our gene-based studies also have limitations for examining the overall mechanism of kidney damage.

## Figures and Tables

**Table 1:** Information and predicted values of five drugs.

Drug	CAS No.	Source	IC_50_ (M)	
			ABC-SC0037	HEK293F
Doxorubicin hydrochloride	25316-40-9	Sigma-Aldrich, MA, USA	1.498 × 10^−5^	6.600 × 10^−6^
(Z)-4-Hydroxytamoxifen	68047-06-3	Tocris, Bristol, UK	1.567 × 10^−5^	5.641 × 10^−5^
Amiodarone hydrochloride	19774-82-4	Sigma-Aldrich	8.229 × 10^−5^	8.336 × 10^−5^
Acetaminophen	103-90-2	Sigma-Aldrich	1.744 × 10^−3^	-
Valproic acid sodium salt	1069-66-5	Sigma-Aldrich	1.429 × 10^−4^	-

IC_50_: half-inhibitory concentration of cell viability.

## Data Availability

Not applicable.
